# Non-*tenera* Contamination and the Economic Impact of *SHELL* Genetic Testing in the Malaysian Independent Oil Palm Industry

**DOI:** 10.3389/fpls.2016.00771

**Published:** 2016-06-21

**Authors:** Leslie C.-L. Ooi, Eng-Ti L. Low, Meilina O. Abdullah, Rajanaidu Nookiah, Ngoot C. Ting, Jayanthi Nagappan, Mohamad A. A. Manaf, Kuang-Lim Chan, Mohd A. Halim, Norazah Azizi, Wahid Omar, Abdul J. Murad, Nathan Lakey, Jared M. Ordway, Anthony Favello, Muhammad A. Budiman, Andrew Van Brunt, Melissa Beil, Michael T. Leininger, Nan Jiang, Steven W. Smith, Clyde R. Brown, Alex C. S. Kuek, Shabani Bahrain, Allison Hoynes-O’Connor, Amelia Y. Nguyen, Hemangi G. Chaudhari, Shivam A. Shah, Yuen-May Choo, Ravigadevi Sambanthamurthi, Rajinder Singh

**Affiliations:** ^1^Malaysian Palm Oil BoardKajang, Malaysia; ^2^Orion Genomics, LLC, St. LouisMO, USA; ^3^Orion Biosains Sdn BhdPuchong, Malaysia; ^4^BALSA Group, St. LouisMO, USA

**Keywords:** oil palm, *tenera*, fruit form, shell, genetic testing

## Abstract

Oil palm (*Elaeis guineensis*) is the most productive oil bearing crop worldwide. It has three fruit forms, namely *dura* (thick-shelled), *pisifera* (shell-less) and *tenera* (thin-shelled), which are controlled by the *SHELL* gene. The fruit forms exhibit monogenic co-dominant inheritance, where *tenera* is a hybrid obtained by crossing maternal *dura* and paternal *pisifera* palms. Commercial palm oil production is based on planting thin-shelled *tenera* palms, which typically yield 30% more oil than *dura* palms, while *pisifera* palms are female-sterile and have little to no palm oil yield. It is clear that *tenera* hybrids produce more oil than either parent due to single gene heterosis. The unintentional planting of *dura* or *pisifera* palms reduces overall yield and impacts land utilization that would otherwise be devoted to more productive *tenera* palms. Here, we identify three additional novel mutant alleles of the *SHELL* gene, which encode a type II MADS-box transcription factor, and determine oil yield via control of shell fruit form phenotype in a manner similar to two previously identified mutant *SHELL* alleles. Assays encompassing all five mutations account for all *dura* and *pisifera* palms analyzed. By assaying for these variants in 10,224 mature palms or seedlings, we report the first large scale accurate genotype-based determination of the fruit forms in independent oil palm planting sites and in the nurseries that supply them throughout Malaysia. The measured non-*tenera* contamination rate (10.9% overall on a weighted average basis) underscores the importance of *SHELL* genetic testing of seedlings prior to planting in production fields. By eliminating non-*tenera* contamination, comprehensive *SHELL* genetic testing can improve sustainability by increasing yield on existing planted lands. In addition, economic modeling demonstrates that *SHELL* gene testing will confer substantial annual economic gains to the oil palm industry, to Malaysian gross national income and to Malaysian government tax receipts.

## Introduction

The *Elaeis* genus comprises two interfertile species, *E. guineensis* of West African origin and *E. oleifera* of Central and South American origin (Zeven, 1965; Cochard et al., 2005). Due to higher oil yield, *E. guineensis* is the predominant species in commercial planting sites, and oil palm cultivation accounts for 45% of edible oil production worldwide. Oil palm has three naturally occurring fruit forms (*dura*, *pisifera*, and *tenera*) which vary in oil yield depending on the presence and thickness of the distinct lignified shell surrounding the fruit kernel. *Dura* fruits have a thick shell (2–8 mm) and produce approximately 5.3 tons of oil per hectare per year. *Pisifera* fruits have no shell, are typically female sterile, and bunches prematurely rot prior to oil production. Crosses of *dura* and *pisifera* palms produce hybrids having *tenera* fruit with a relatively thin shell (0.5–3 mm) surrounded by a distinct fiber ring and can produce from 7.4 tons (Hartley, 1988) to as high as 13.6 tons of oil per hectare per year, as seen in elite individuals (Sharma and Tan, 1999). Therefore, *tenera* hybrids produced by selection of maternal (*dura*) and paternal (*pisifera*) pools are the basis for commercial palm oil production throughout southeast Asia (Rajanaidu et al., 2000).

The shell fruit form phenotype displays co-dominant monogenic inheritance (Beirnaert and Vanderweyen, 1941). Our previous identification of the *SHELL* gene and two mutations responsible for *tenera* and *pisifera* fruit forms explained the single gene heterosis exhibited in *tenera* palms (Singh et al., 2013a). The discovery was recently independently verified and converted to a PCR based assay (Ritter et al., 2015). SHELL is a type II MADS-box transcription factor homologous to *Arabidopsis* SEEDSTICK (STK) and rice OsMADS13, which are members of transcription factor networks controlling differentiation of the ovule, seed and lignified endocarp in *Arabidopsis* (Favaro et al., 2003; Pinyopich et al., 2003; Dinneny and Yanofsky, 2005) and ovule differentiation and female fertility in rice (Dreni et al., 2007). MADS-box proteins function through heterodimerization with other MADS-box family members, and as predicted by homology, wild-type SHELL heterodimerizes with the rice SEPALLATA MADS-box protein, OsMADS24, in yeast two-hybrid assays (Singh et al., 2013a). The *sh^MPOB^* mutant allele, identified among descendants of the Nigerian *tenera* accession T128, is a T-to-C polymorphism that results in a leucine-to-proline amino acid change within the highly conserved MADS-box domain. The *sh^AVROS^* mutant allele, identified within a multigenerational pedigree spanning five-decades segregating for the Congo-derived AVROS *pisifera* allele, is an A-to-T polymorphism resulting in a lysine-to-asparagine substitution, which is two amino acids carboxy-terminal to the *sh^MPOB^* amino acid change (Singh et al., 2013a). Both mutations occur within the α-helical structure, characteristic of all MADS-box domains, which is involved in both heterodimerization and DNA binding. Palms producing thick-shelled *dura* fruits are homozygous for the wild-type (*Sh^DeliDura^*) nucleotide at each of the two variant nucleotide positions. Heterozygosity for either the *sh^MPOB^* or *sh^AVROS^* alleles (*Sh^DeliDura^/sh^MPOB^* or *Sh^DeliDura^/sh^AVROS^*) results in palms producing thin-shelled *tenera* fruits. Palms homozygous for either mutation or heteroallelic for both mutations (a *sh^AVROS^* mutation on one chromosome and a *sh^MPOB^* mutation on the other) produce shell-less *pisifera* fruits. Cases of heteroallelic *pisifera* palms indicated that *sh^AVROS^* and *sh^MPOB^* alleles do not complement each other, thus confirming the identity of the *SHELL* gene (Singh et al., 2013a).

The intention of the oil palm industry is to plant only high-yielding *tenera* hybrids in production fields. However, non-*tenera* contamination can arise due to several reasons, including unintentional use of pollen from a non-*pisifera* palm, self-pollination of *dura* parental palms, open pollination of *dura* parental palms by surrounding *dura* palms, and imprecise selection of seeds or seedlings (Corley, 2005). The phenotypic identification of non-*tenera* contaminant palms is based on the observation of a cross-section of mature fruit. Since fruiting first occurs 3–4 years after field planting, the replacement of contaminants is not economical due to the large size of palms at this stage. With the discovery of *SHELL* and the mutations responsible for fruit form, DNA-based fruit form prediction is possible in nurseries prior to field planting.

We report the application of *SHELL* genetic testing in the assessment of non-*tenera* contamination rates in planting and nursery sites throughout Malaysia. Contamination rates are substantially higher than the <1% theoretically achievable through careful control of pollination (Corley, 2005). DNA sequencing identified three novel mutant alleles of *SHELL*, each resulting in the *tenera* fruit form when heterozygous. Like the *sh^AVROS^* and *sh^MPOB^* mutations, each mutant allele is a missense mutation resulting in substitution of a conserved amino acid within the SHELL MADS-box domain, indicating that all five *SHELL* mutations share a common or closely related molecular mechanism impacting oil palm fruit form.

We also constructed a 48-parameter, 4-stage economic model to determine the losses incurred by the inadvertent cultivation of non*-tenera* palms in Malaysia. The findings suggest that comprehensive DNA-based screening and removal of non-*tenera* contaminants at the nursery will increase yield and result in substantial economic gains while optimizing use of existing planted area – a positive step toward sustainability.

## Materials and Methods

### Sampling of Independent Planting Sites and Independent Nurseries

Non-*tenera* contamination rates were assessed in representative independent planting sites surrounding the Malaysian Palm Oil Board’s (MPOB’s) 6 research stations, located in Peninsular Malaysia (Teluk Intan, Kluang, Keratong, Hulu Paka), Sarawak (Sessang) and Sabah (Lahad Datu). Non-*tenera* contamination rates were also assessed in three independent nursery sites in each of seven sampling sub-regions encompassing the six geographical locations selected for the independent planters. The seven sampling sub-regions include Perak, Selangor, Negeri Sembilan, Melaka, Johor, and Sarawak. In total, approximately 200 palms from each of 36 independent planting sites (*n* = 6,272) and from each of 21 nursery sites (*n* = 3,952) were randomly selected and genotyped by allele-specific PCR.

### PCR Based Genetic Testing

In a total of 10,224 samples, genotypes at the *sh^AVROS^* and *sh^MPOB^* variant nucleotide positions were determined by SureSawit^TM^ *SHELL* Kit (Orion Biosains^1^). Approximately 20 ng of genomic DNA were used as template for each of four independent allele-specific PCR reactions: (i) exon 1 specific primers in which one primer overlapped the *sh^MPOB^* variant nucleotide with the wild-type base and amplified only the wild-type allele at the variant nucleotide position, (ii) exon 1 specific primers in which one primer overlapped the *sh^MPOB^* variant nucleotide with the *sh^MPOB^* base and amplified only the *sh^MPOB^* allele at the variant nucleotide position, (iii) exon 1 specific primers in which one primer overlapped the *sh^AVROS^* variant nucleotide with the wild-type base and amplified only the wild type allele at the variant nucleotide position, and (iv) exon 1 specific primers in which one primer overlapped the *sh^AVROS^* variant nucleotide with the mutant base and amplified only the *sh^AVROS^* allele at the variant nucleotide position. PCR wells were scored for amplification at end point by SYBR Green emission. Genotypes were scored based on the amplification pattern across the four reactions. Samples that are wild-type at both variant positions (*Sh^DeliDura^/Sh^DeliDura^*) amplify in reactions (i) and (iii) only. *Sh^DeliDura^/sh^MPOB^* samples amplify in reactions (i), (ii), and (iii) only. *Sh^DeliDura^/sh^AVROS^* samples amplify in reactions (i), (iii), and (iv) only. *sh^AVROS^/sh^AVROS^* samples amplify in reactions (i) and (iv) only. *sh^MPOB^/sh^MPOB^* samples amplify in reactions (ii) and (iii) only. Finally, heteroallelic *sh^AVROS^/sh^MPOB^* samples amplify in all four reactions. Primer sequences and PCR conditions are provided in Supplementary Table S1.

### DNA Sequencing

A total of 1,132 palm or seedling samples were genotyped by allele-specific PCR as wild-type at both the *sh^AVROS^* and *sh^MPOB^* variant nucleotide positions. Although, these palms or seedlings would be predicted to be *dura* phenotype based on the genotypes at the *sh^AVROS^* and *sh^MPOB^* positions alone, they could, in fact, be *tenera* phenotype due to *SHELL* mutations independent of the *sh^AVROS^* and *sh^MPOB^* mutations. Therefore, exon 1 of *SHELL* was PCR amplified using flanking intronic primers (Singh et al., 2013a) and Sanger sequenced. Primer sequences were confirmed to be unique in the reference *pisifera* genome and free of polymorphic nucleotides (Singh et al., 2013b). *SHELL* exon 1 was amplified from 20 ng of purified genomic DNA under standard PCR amplification conditions (Singh et al., 2013a). Amplicons were treated with exonuclease I and shrimp alkaline phosphatase to remove unincorporated primers and deoxynucleotides. An aliquot of each amplicon was Sanger sequenced (ABI 3730) using an amplicon specific primer. All sequencing data was aligned to the reference *pisifera* genome sequence, and data was analyzed to determine the genotype at each exon 1 base. *SHELL* exons 2–7 were similarly Sanger sequenced in a subset of samples. Exons 3–6 were amplified using an intron 2 forward primer and an intron 6 reverse primer. Exons 2 and 7 were separately amplified using intronic primers flanking each exon. All primer sequences and PCR conditions are provided in Supplementary Table S1.

### Genotype/Phenotype Comparisons

To determine concordance of fruit form phenotype with *SHELL* genotype, 512 previously unanalyzed samplings were phenotyped by visual inspection of shell thickness in cross-sectioned ripe fruits and genotyped by Sanger sequencing of *SHELL* exon 1, as described above. Phenotyping and genotyping were performed by different individuals. Individuals involved in phenotyping were blinded to genotyping calls, and individuals involved in genotyping were blinded to phenotyping calls. Genotypes and phenotypes were compared only after each set of calls was finalized. To address initial genotype/phenotype discordances, 15 discordant palms, as well as 15 palms that were concordant in the initial analysis, were sampled a second time for independent blinded phenotyping and genotyping as described above.

### Economic Impact Modeling of Comprehensive SHELL Genetic Testing

To assess the economic impact of *SHELL* genetic testing on the Malaysian oil palm industry, Malaysian gross national income (GNI) and tax revenues of the Malaysian government, a 48-parameter, four-stage economic model that involves breeders, nurseries, planting sites and mills was constructed (**Table 4**). In the model, two scenarios were compared – a baseline scenario representing the current state of the independent planters and a *SHELL* gene testing scenario, which forecasts changes in the production of crude palm oil (CPO), palm kernel oil (PKO), and palm kernel cake (PKC) in the deployment of comprehensive DNA testing and culling at the nursery stage. Contamination rates utilized for economic modeling were based on weighted averages of observed contamination in the surveyed independent planting sites, as these values represent the closest approximation of contamination currently in production fields. Observed contamination rates within each planting region were weighted according to the hectare size of planting area for each region, resulting in the *pisifera* (2.8%) and *dura* (8.1%) contamination rates utilized in the baseline scenario. Note that non-weighted contamination rates are reported in **Table 1**. Assuming that comprehensive *SHELL* gene testing would enable the molecular based identification and subsequent culling of all contaminant *pisifera* and *dura* palms at the nursery stage, we modeled 100% *tenera* rates in the selected sites in the *SHELL* gene testing scenario.

**Table 1:** Non-*tenera* contamination by region.

Region^a^	No. sites surveyed	Palms genotyped	*tenera*^b^ (%)	*dura*^c^ (%)	*pisifera*^d^ (%)	Unadjusted non-*tenera* contamination^e^ (%)
1	6	1,132	88.9	7.4	3.7	11.1
2	6	1,133	89.1	9.7	1.1	10.9
3	6	882	96.4	2.8	0.8	3.6
4	6	1,092	97.5	2.3	0.2	2.5
5	3	539	92.8	7.1	0.2	7.2
6	3	571	79.7	20.1	0.2	20.3
7	3	576	76.9	22.7	0.3	23.1
8	3	571	84.2	15.6	0.2	15.8
9	3	550	95.8	3.8	0.4	4.2
10	6	921	91.6	8.1	0.2	8.4
11	6	1,112	81.0	11.8	7.2	19.0
12	3	572	88.5	11.4	0.2	11.5
13	3	573	94.1	5.6	0.3	5.9
**Total**	57	10,224	89.3	9.2	1.5	10.7

*^a^Regions within Peninsular Malaysia (1–9) and Sabah and Sarawak (10–13) were surveyed.*

*^b^*Tenera* genotypes include *Sh^*DeliDura*^/sh^*AVROS*^, Sh^*DeliDura*^/sh^*MPOB*^, Sh^*DeliDura*^/sh^*MPOB2*^, Sh^*DeliDura*^/sh^*MPOB3*^*, or *Sh^*DeliDura*^/sh^*MPOB4*^*.*

*^c^*Dura* genotypes are wild-type at all five variant nucleotide positions.*

*^d^*Pisifera* genotypes include *sh^*AVROS*^/sh^*AVROS*^, sh^*MPOB*^/sh^*MPOB*^*, or *sh^*AVROS*^/sh^*MPOB2*^.**

*^e^Note that national weighted average contamination rate adjusted for the regional planting area is 10.9%.*

Monte Carlo simulation was used to mimic variation around four key fruit composition parameters including fruit to bunch, mesocarp to fruit, shell to fruit, and kernel to fruit for *tenera* and *dura* palms, where 1,000 values were selected at random from normal distributions of each parameter for each fruit form for each year in the 60-years simulation (**Table 4**). For *pisifera* palms in the baseline scenario, we assumed these trees produced no fruit and were a complete loss.

We held planted area managed by independent planters at reported 2015 levels throughout the simulation (0.809 M HA, representing 15% of the 5.39 M HA of total planted area in Malaysia), and assumed an average planting density of 143 palms per hectare. Assumptions surrounding the age structure and productivity of palms in year 0 of the simulation are summarized in **Table 4**, as are mill extraction efficiencies and other parameters.

The mass of CPO, PKO, and PKC for the baseline and *SHELL* testing scenarios were computed from the following mass balance equations:

(i) FFB (fresh fruit bunch) = EFB (empty fruit bunch) + PF (palm fruit);
(ii) PF (palm fruit) = Msc (mesocarp) + PK (kernel) + PKS (palm kernel shell);
(iii) Msc (mesocarp) = CPO (crude palm oil) + MF (mesocarp fruit fiber) + POME_MSC_ (fraction of palm oil mill eﬄuent from mesocarp);
(iv) PK (kernel) = PKO (palm kernel oil) + PKC (palm kernel cake) + WW_PK_ (fraction of palm kernel mill waste water from kernel).

Substituting terms of equation (i) with equations (ii), (iii), and (iv) yields equation (v):

(v) FFB = EFB + CPO + MF + POME_MSC_ + PKO + PKC + WW_PK_ + PKS.

To determine the selling price (P) of CPO and PKO at a future date (i.e., future month A), a regression of monthly closing prices denominated in Malaysian Ringgit from January 1995 through April 2015 for CPO^2^ and from January 1996 through April 2015 for PKO^3^, was computed and the following exponential trend equations and effective inflation rates were determined and used in the model (historic prices for PKO denominated in Malaysian Ringgit were not available in 1995):

P_CPO_(month A) = 1107.9 × e(0.0039 × B); an effective inflation rate of 4.79%
P_PKO_(month A) = 1602.5 × e(0.0037 × B); an effective inflation rate of 4.54%

where B equals the number of months from January 1995 to Month A.

A price inflation rate of 4.79% (based on the effective inflation rate of CPO) and a bond rate of 3.55% were used for time value of money corrections, an economic multiplier of 1.511 (as determined for oil palm primary products in Malaysia; Bekhet, 2011) was used to convert industry economic gains into increases in GNI, and a weighted average tax rate of 25% (the standard Malaysian corporate income tax rate since 2009) applied to increases in GNI was used to compute increases in Malaysian government tax receipts. The costs of sampling and culling labor at the nursery, shipping sampled material to a centralized laboratory and the costs of *SHELL* allele testing were not included in the model.

## Results

### Non-*tenera* Contamination Trial Design

Adult palms and seedlings were selected among six planting sites and three nearby independent nurseries within multiple geographical regions throughout Peninsular Malaysia, Sabah and Sarawak (**Table 1**). The number of sampled palms per region ranged from 539 to 1,133, for a total of 10,224 independent palms. To ensure that the palms sampled from each site were representative, plants were randomly selected from several operational areas within each site. *SHELL* genotypes for two previously described mutations (*sh^AVROS^* and *sh^MPOB^*) were determined by allele-specific PCR assays designed to amplify either the *dura* or *pisifera* allele (see Materials and Methods). In addition to *sh^AVROS^* and *sh^MPOB^* mutant alleles, **Table 1** includes three novel *SHELL* mutations described below.

### Non-*tenera* Contamination

Palms were classified as genetically non-*tenera* if the genotype was either homozygous wild type at both variant nucleotide positions and therefore *dura* fruit form, or homozygous or heteroallelic for *sh^AVROS^* and/or *sh^MPOB^* mutant alleles (*sh^AVROS^/sh^AVROS^*, *sh^MPOB^/sh^MPOB^*, or *sh^AVROS^/sh^MPOB^*) and therefore *pisifera* fruit form. Palms that were homozygous or heteroallelic for any of three novel *SHELL* gene mutations, described below, were also classified as non-*tenera.* Palms identified as genetically *dura* were confirmed by DNA sequencing to have no other *SHELL* gene mutations that could have caused the palm to be a *tenera* phenotype palm (see Materials and Methods). The unadjusted non-*tenera* contamination across all palms sampled was 10.7% [95% confidence interval (CI) 10.1–11.9%; **Table 1**]. The average contamination rates from each region were then weighted by each region’s respective total oil palm planted area resulting in a national weighted average contamination rate in independent planting sites of 10.9% (2.8% *pisifera* and 8.1% *dura*).

**FIGURE 1:**
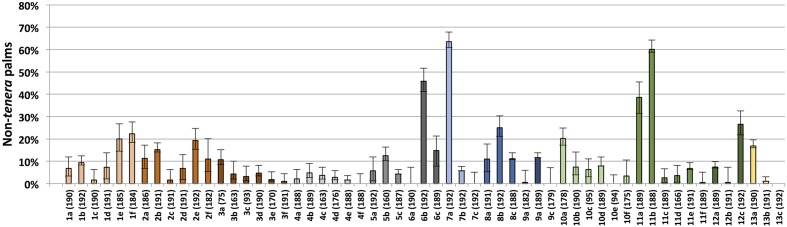
**Non-*tenera* contamination rates within each individual sampled site**. Palms and nursery seedlings genotyped as either wild-type (*dura*), homozygous mutant for any of the five identified *SHELL* gene MADS box mutations (*pisifera*) or heteroallelic for any two of the five mutations (*pisifera*) were classified as non-*tenera*. On the x-axis, numbers 1–13 indicate sampled regions according to **Table 1**. Letters represent each independent site sampled within the indicated region. Numbers in parentheses indicate the number of palms or seedlings genotyped per region/site. Error bars indicate 95% confidence intervals.

Both *dura* and *pisifera* contamination were observed in each region with *dura* contamination exceeding that of *pisifera* in each case (**Table 1**). This finding indicates that contamination is not completely explained by self or open pollination of *dura* palms by *dura* pollen (which would yield only *dura* seeds) or by unintentional pollination of *dura* palms by *tenera* pollen (which would yield *dura* or *tenera* seeds), as *pisifera* seeds could only be produced from *tenera* × *pisifera* or *tenera* × *tenera* crosses. Regional unweighted non-*tenera* contamination ranged from 2.5% (Region 4) to 23.1% (Region 7). There was significant variation in non-*tenera* contamination between different sites within a given region, with the exception of the two lowest overall contamination sites (Regions 3 and 4; **Figure 1**). Furthermore, individual sites with very high contamination rates were identified, for example, site 7a with 63.5% (95% CI 56.5–70.1%) non-*tenera* palms and site 11b with 60.1% (95% CI 52.9–67.0%) non-*tenera* palms. Only 6 of 57 sites had no detectable non-*tenera* palms (**Figure 1**). Palms of all age groups sampled (from less than 5 years to greater than 10 years after planting) showed non-*tenera* contamination, indicating that current controlled pollination practices have not overcome the problem.

### Identification of Novel Mutant Alleles of *SHELL*

We previously identified two independent but closely related mutations within the *SHELL* MADS-box domain (*sh^AVROS^* and *sh^MPOB^*) that each results in the *tenera* fruit form phenotype when heterozygous and the *pisifera* fruit form phenotype when homozygous or heteroallelic with each other (Singh et al., 2013a). However, recognizing that additional mutant alleles of *SHELL* could exist in commercial material, exon 1 of the *SHELL* gene (encoding the entire MADS-box domain) was sequenced in each of the DNA samples with a wild-type genotype at both the *sh^AVROS^* and *sh^MPOB^* nucleotide variant positions (see Materials and Methods).

**FIGURE 2:**
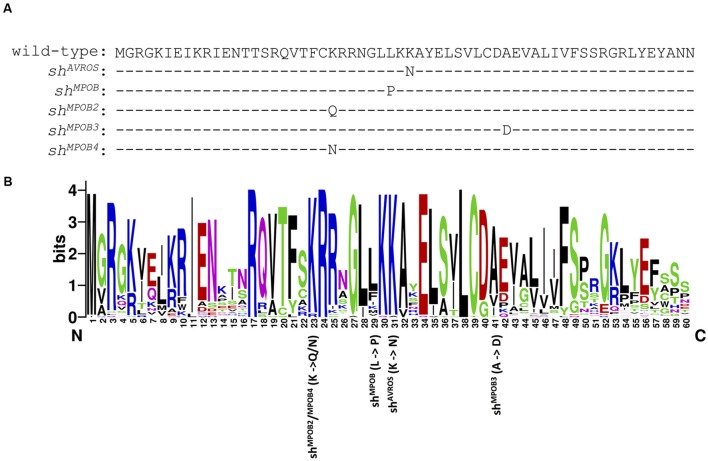
***SHELL* MADS box domain mutations associated with fruit form phenotype**. **(A)** The amino acid sequence of the wild-type (*dura*) SHELL MADS box domain is shown at the top. Amino acid substitutions encoded by the *sh^AVROS^, sh^MPOB^, sh^MPOB2^, sh^MPOB3^*, and *sh^MPOB4^* mutations are each indicated below the wild-type amino acid sequence. **(B)** Logo plot of amino acid conservation among 51 independent oil palm MADS box gene predictions within the oil palm reference genome sequence. The positions of substituted amino acids encoded by the five *SHELL* mutations are indicated below the plot. The *sh^MPOB2^* (K-to-Q) and *sh^MPOB4^* (K-to-N) mutations alter the same amino acid (position 23 in the Logo plot). The *sh^MPOB^*, *sh^AVROS^*, and *sh^MPOB3^* mutations alter amino acid positions 29, 31, and 41 in the Logo plot, respectively. Each mutation results in alteration of the most prevalent amino acid to an amino acid not utilized in any oil palm MADS box gene prediction at that position.

Among 1,132 palms genotyped as homozygous wild-type at the *sh^AVROS^* and *sh^MPOB^* nucleotide positions, 235 (20.8%) were heterozygous for one of three novel non-synonymous nucleotide variants within the MADS-box coding region (**Table 2**; **Figure 2A**), and no palms were found to be homozygous for these variants. Two variants [*sh^MPOB2^* (lysine-to-glutamine) and *sh^MPOB4^* (lysine-to-asparagine)] result in the substitution of the same conserved lysine six residues amino-terminal to the *sh^MPOB^* leucine-to-proline position. The *sh^MPOB3^* variant results in an alanine-to-aspartate substitution 10 residues carboxy-terminal to the *sh^AVROS^* lysine-to-asparagine position (**Figure 2**). The three novel mutant *SHELL* alleles varied in frequency. The *sh^MPOB2^* variant was detected in 1.4% of the 10,224 palms analyzed (**Table 2**), and was present in 7 of the 13 geographic regions at rates ranging from 0.1% (Region 1) to 6.4% (Region 10). The *sh^MPOB3^* variant was detected in 7 of 13 regions, at a rate ranging from 0.3% (Region 7) to 2.1% (Region 1), representing 0.8% of all palms analyzed. Finally, the *sh^MPOB4^* variant was detected in only two palms (in Region 1 and Region 3), representing 0.02% of all palms analyzed. Given the conservation of these residues within the MADS-box domain motif (**Figure 2B**) and the proximity to the mutations previously shown to determine fruit form phenotype (Singh et al., 2013a), we hypothesized that these variants represent novel mutant alleles of *SHELL* that also control oil palm shell fruit form. Note that the non-*tenera* contamination rates described in **Table 1** and **Figure 1** were calculated based on all five *SHELL* gene variants. All seven exons of *SHELL* were sequenced in a panel of 112 palms that were homozygous wild-type at all five variant nucleotide positions. While three synonymous single nucleotide polymorphisms (SNPs) were identified within exon 7, these would not be predicted to impact fruit form as they code for the wild-type amino acid. One non-synonymous SNP resulting in a conservative glutamate-to-aspartate substitution 12 amino acids from the SHELL carboxy terminus was identified.

**Table 2:** Novel *SHELL* alleles.

	Potential *dura* contaminants^a^	*sh^MPOB2^*	*sh^MPOB3^*	*sh^MPOB4^*
Region 1	110	1 (0.1%)^b^	24 (2.1%)	1 (0.1%)
Region 2	126	–	16 (1.4%)	–
Region 3	32	–	5 (0.6%)	1 (0.1%)
Region 4	36	–	11 (1.0%)	–
Region 5	37	1 (0.2%)	–	–
Region 6	114	18 (3.2%)	–	–
Region 7	129	12 (2.1%)	2 (0.3%)	–
Region 8	89	5 (0.9%)	–	–
Region 9	20	–	–	–
Region 10	145	59 (6.4%)	11 (1.2%)	–
Region 11	199	52 (4.7%)	16 (1.4%)	–
Region 12	65	–	–	–
Region 13	30	–	–	–
**Total**	1,132	148 (1.4%)	85 (0.8%)	2 (0.02%)

*^a^Number of palms genotyped as wild-type at both the *sh^*AVROS*^* and *sh^*MPOB*^* variant nucleotide positions.*

*^b^Integer value represents number of palms per site heterozygous for the indicated nucleotide variant, and parentheses denote the percentage of the indicated heterozygous palms among all palms analyzed per site.*

To demonstrate that the three novel mutant *SHELL* alleles conferred the shell fruit form trait, adult palms from populations in which the alleles were detected were sampled and scored for fruit form. In total, 512 sampled palms with mature fruit bunches were visually phenotyped and DNA sequenced (see Materials and Methods). In an initial genotype-by-phenotype comparison, 97.1% of genotypes matched the predicted phenotype, assuming that all five mutant alleles result in *tenera* phenotype when heterozygous and *pisifera* phenotype when homozygous. A 2.9% discordance rate is within the accuracy norms of visual phenotyping in plantation settings (Singh et al., 2013a). The 15 discordant palms included nine *Sh^DeliDura^/sh^AVROS^* palms and one *Sh^DeliDura^/sh^MPOB2^* palm phenotyped as *dura*, two *sh^AVROS^*/*sh^AVROS^* palms and one *Sh^DeliDura^*/ *Sh^DeliDura^* palm phenotyped as *tenera* and 2 *Sh^DeliDura^/sh^AVROS^* palms phenotyped as *pisifera*. There were no discordances in which a genotypically *pisifera* palm was phenotyped as *dura*, or *vice versa*, as these phenotypes are the most easily discriminated by visual inspection of shell thickness. However, in addition to a low rate of visual phenotyping inaccuracy, discordances could arise due to inaccuracies of genotyping as well as due to potential sample collection and processing errors that may arise, especially under conditions where 100–1000s of samples are collected in field settings. To address this, the 15 discordant palms, as well as 15 palms that were concordant in the initial analysis, were sampled a second time for independent blinded phenotyping and genotyping (see Materials and Methods). In this independent round of sampling, no phenotype or genotype changes occurred among the 15 originally concordant palms, and therefore these remained genotype-by-phenotype concordant. However, among the 15 originally discordant palms, all 15 were either scored as a different phenotype than in the first sampling (six palms) or genotyping of the repeated sampling yielded a different genotype than the first sampling (nine palms), and these changes resolved each of the 15 discordances. These results suggest that the very low rate of discordances was due to a combination of phenotyping and sample processing inaccuracies. First, all genotyping in this genotype-by-phenotype comparison was performed by Sanger DNA sequencing, which has a very low error rate. Second, if genotyping inaccuracies were the cause of discordances, then genotype calls should have changed at a similar rate among the initially concordant and discordant palm sets. However, 60% of initial discordant palms changed genotype upon second sampling compared to no palms changing genotype within the initially concordant set (*p* < 0.0003, chi squared test). To further support this conclusion, we performed three independent PCR amplification and Sanger sequencing assays on each of the 30 original sampling DNA preps, as well as each of the 30 resampled DNA preps. In every case (180 independent assays), genotype calls were consistent between replicated assays of the same sample and agreed with the original genotype call of the same prep, thus demonstrating the accuracy of genotype calls.

**FIGURE 3:**
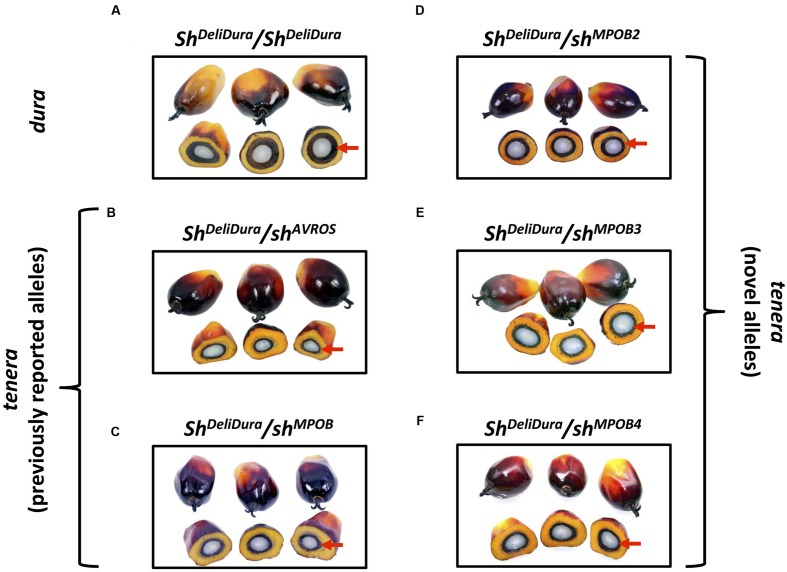
**Fruit form phenotypes**. Photographs of three whole fruits (top) and three cross-sectioned fruits (bottom) from a wild-type *dura* palm **(A)**, and from *Sh^DeliDura^/sh^AVROS^* **(B)**, *Sh^DeliDura^/sh^MPOB^* **(C)**, *Sh^DeliDura^/sh^MPOB2^* **(D)**, *Sh^DeliDura^/sh^MPOB3^* **(E)**, or *Sh^DeliDura^/sh^MPOB4^* **(F)** *tenera* palms are shown. Arrows indicate the thick shell of *dura* fruit **(A)** and the thin shell of *tenera* fruits from palms with different heterozygous mutant alleles **(B–F)**.

After resolution of rare discordances, the five *SHELL* MADS box domain mutations accounted for 100% of observed *tenera* and *pisifera* phenotypes (**Table 3**; **Figure 3**). Among the 422 phenotypically *tenera* palms, 385 were heterozygous *sh^AVROS^/Sh^DeliDura^*, two were heterozygous *sh^MPOB^/Sh^DeliDura^*, two were heterozygous *sh^MPOB2^/Sh^DeliDura^*, 31 were heterozygous *sh^MPOB3^/Sh^DeliDura^*, and two were heterozygous *sh^MPOB4^/Sh^DeliDura^*. All 56 phenotypically *dura* palms were wild-type at each of the five variant positions. Finally, among 34 phenotypically *pisifera* palms, 33 were homozygous *sh^AVROS^/sh^AVROS^* and one was heteroallelic with the *sh^MPOB2^* allele on one chromosome and the *sh^AVROS^* allele on the other. This heteroallelic *pisifera* palm suggests that *sh^MPOB2^* does not complement the *sh^AVROS^* allele, thus confirming that the *sh^MPOB2^* mutation (and, likely, the *sh^MPOB4^* mutation, as it involves substitution of the same amino acid) functionally determines oil palm shell fruit form. Furthermore, the 100% concordance between heterozygosity for *sh^MPOB3^* and *tenera* phenotype strongly supports the conclusion that this allele is functionally equivalent to the other four mutant alleles. The finding that all *tenera* (*n* = 442) and all *pisifera* (*n* = 34) phenotype palms could be explained by heterozygous (*tenera*) or homozygous/heteroallelic (*pisifera*) mutations involving the five *SHELL* MADS box domain mutations indicates that the single conservative missense amino acid substitution detected in exon 7 likely does not impact shell fruit form phenotype. While additional mutant alleles impacting fruit form may be present in other oil palm populations, the data confirm that the three novel *SHELL* alleles affect fruit form and suggest that these five mutations are responsible for at least the vast majority of *tenera* and *pisifera* phenotypes within this wide sampling of planting sites and nurseries.

**Table 3:** Concordance of *SHELL* genotype and fruit form phenotype.

		Observed phenotypes^b^		
Predicted phenotypes^a^	Genotypes	*tenera*	*pisifera*	*dura*
*tenera*	*Sh^DeliDura^/sh^AVROS^*	385	–	–
	*Sh^DeliDura^/sh^MPOB^*	2	–	–
	*Sh^DeliDura^/sh^MPOB2^*	2	–	–
	*Sh^DeliDura^/sh^MPOB3^*	31	–	–
	*Sh^DeliDura^/sh^MPOB4^*	2	–	–

*pisifera*	*sh^AVROS^/sh^AVROS^*	–	33	–

	*sh^AVROS^/sh^MPOB2^*	–	1	–
*dura*	*Sh^DeliDura^/Sh^DeliDura^*	–	-	56

*^a^Fruit form phenotypes predicted by the indicated genotype*.

*^b^Fruit form phenotypes determined by visual inspection*.

### Economic Impact Modeling of Comprehensive *SHELL* Genetic Testing

**FIGURE 4:**
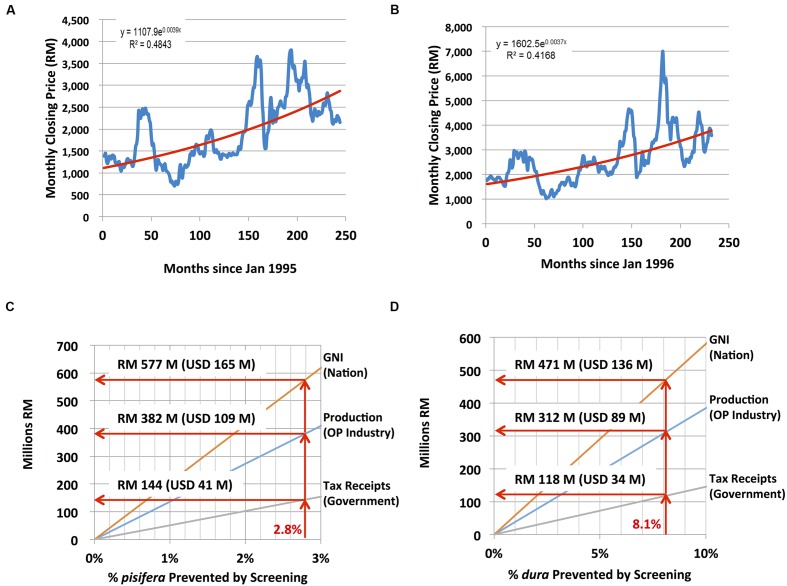
**Economic impact modeling of comprehensive *SHELL* genetic testing**. To determine selling price of CPO **(A)** and PKO **(B)** at a future date, monthly closing prices (in Malaysian Ringgit, RM) are plotted from January 1995 through April 2015 **(A)** and from January 1996 through April 2015 (historic prices for PKO denominated in Malaysian Ringgit were not available in 1995); **(B)**. A regression of monthly closing prices was computed as described in section “Materials and Methods.” Annual economic impact of preventing *pisifera* **(C)** and *dura* **(D)** contamination in independent planting sites at steady state. RM to U.S. dollar (USD) conversions are based on an average exchange rate over the past 20 years of 3.5RM/USD.

**FIGURE 5:**
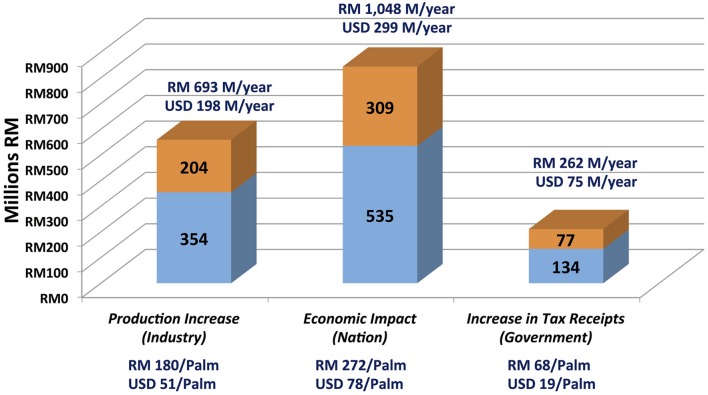
**Annual economic impact of *SHELL* genetic testing on the oil palm industry, gross national income and the Malaysian government**. Calculated annual increases (Malaysian Ringgit, RM) are plotted as proportions obtained by genetic screening based prevention of *dura* (blue) and *pisifera* (orange) contamination among independent planting sites. Gains per individual palm screened prior to commercial planting including *tenera* and non-*tenera* palms are indicated below each column. RM to U.S. dollar (USD) conversions are based on an average exchange rate over the past 20 years of 3.5RM/USD.

Nursery stage DNA testing of the estimated 3.9 million palms planted at independent planting sites each year would enable nursery operators to cull contaminant palms before they are field planted. To assess the economic impact of *SHELL* genetic testing in the independent sector, a 48-parameter, four-stage economic model that involves breeders, nurseries, planting sites and mills was constructed (**Table 4**, see Materials and Methods). The monthly closing prices for CPO and PKO and the fit of the regression curves for CPO and PKO are plotted in **Figures 4A,B**, respectively, where the regression equations were able to explain 48.43 and 41.68% of the historical pricing data, and regression predictions were used for future CPO and PKO pricing in the model (see Materials and Methods). The economic model determined two curves, which compute the annual gains at steady state excluding sampling, testing and culling costs (i) to the oil palm industry, (ii) to Malaysian GNI, and (iii) to the Malaysian government in the form of increased tax revenues. The gains are determined as a function of the weighted average national independent planting site *pisifera* (**Figure 4C**) and *dura* (**Figure 4D**) contamination prevented in the *SHELL* gene screening scenario (see Materials and Methods). The *SHELL* gene screening scenario would increase GNI, industry income, and government tax income as low yielding contaminant palms are replaced by high yielding *tenera* palms. At steady state, *SHELL* gene DNA testing in the independent sector alone would add Malaysian Ringgit (RM) 1.05 billion to Malaysian GNI annually by comprehensive screening (or RM 272 in gains per screened palm - including all *tenera* and non-*tenera* palms tested), RM 0.693 BN of increased production annually to oil palm industry members (or RM 180 per screened palm) and RM 0.26 BN of increased tax receipts annually (or RM 68 in new taxes per screened palm; **Figure 5**). Using the average RM to USD exchange rate over the past 20 years of 3.5, *SHELL* gene DNA testing would add ∼$300M USD to Malaysian GNI annually (or $77.71 USD per screened palm), $198M USD of increased production annually to oil palm industry members (or $51.42 USD per screened palm), and ∼$75M USD of increased tax receipts annually (or $19.43 USD per screened palm; **Figure 5**).

**Table 4:** Parameters for Economic Impact Model.

Definition		Value
**Contamination rates:**		
Initial fraction of Tenera		89.10%
Initial fraction of Dura		8.10%
Initial fraction of Pisifera		2.80%
Final fraction of Tenera		100.00%
Final fraction of Dura		0.00%
Final fraction of Pisifera		0.00%
**Plantation:**		
Number of trees per hectare		143
Total planted area (in HA)		5,392,235
Fraction cultivated by Ind. smallholders		15.00%
Total planted area of Ind. smallholders (in HA)		808,835
**Age structure and productivity:**		
Initial fraction of immature palms (age < 4)		13.00%
Initial fraction of young palms (age 5–8)		17.00%
Initial fraction of prime palms (age 9–18)		35.00%
Initial fraction of aging palms (age 19–24)		20.00%
Initial fraction of old palms (age 25–30)		15.00%
Productivity – young palms (MT FFB/HA/YR)(D&T)		15
Productivity – prime palms (MT FFB/HA/YR)(D&T)		23
Productivity – aging palms (MT FFB/HA/YR)(D&T)		17
Productivity – old palms (MT FFB/HA/YR)(D&T)		15

**Definition**	**Parameter**	**Value**

**Milling:**		
CPO mill extraction efficiency	ECPO	86.00%
PKO mill extraction efficiency	EPKO	97.00%
**Valuation:**		
Initial CPO price (in RM)	PCPO	2,869
Initial PKO price (in RM)	PPKO	3,781
Initial PKC price (in RM)	PPKC	370
CPO price inflation rate	RI	4.79%
Bond rate for Malaysia	RB	3.55%
MY corporate income tax rate	Tax	25.00%
MY econ multiplier (oil palm primary products)	M	1.511
**Fruit composition (mass ratios):**		
Tenera: fruit to bunch (Average)	PF/FFB(T)(Average)	0.649
Tenera: fruit to bunch (SD)	PF/FFB(T)(SD)	0.041
Dura: fruit to bunch (Average)	PF/FFB(D)(Average)	0.612
Dura: fruit to bunch (SD)	PF/FFB(D)(SD)	0.038
Tenera: mesocarp to fruit (Average)	Msc/PF(T)(Average)	0.865
Tenera: mesocarp to fruit (SD)	Msc/PF(T)(SD)	0.027
Dura: mesocarp to fruit (Average)	Msc/PF(D)(Average)	0.612
Dura: mesocarp to fruit (SD)	Msc/PF(D)(SD)	0.026
Tenera: shell to fruit (Average)	PKS/PF(T)(Average)	0.07
Tenera: shell to fruit (SD)	PKS/PF(T)(SD)	0.008
Dura: shell to fruit (Average)	PKS/PF(D)(Average)	0.306
Dura: shell to fruit (SD)	PKS/PF(D)(SD)	0.026
Tenera: kernel to fruit (Average)	PK/PF(T)(Average)	0.065
Tenera: kernel to fruit (SD)	PK/PF(T)(SD)	0.005
Dura: kernel to fruit (Average)	PK/PF(D)(Average)	0.082
Dura: kernel to fruit (SD)	PK/PF(D)(SD)	0.01
Tenera: CPO content of mesocarp (Average)	CPO/Msc(T)(Average)	0.55
Dura: CPO content of mesocarp (Average)	CPO/Msc(D)(Average)	0.55
Tenera: PKO content of kernel (Average)	PKO/PK(T)(Average)	0.47
Dura: PKO content of kernel (Average)	PKO/PK(D)(Average)	0.47

*MT, metric tons; FFB, fresh fruit bunch; HA, hectare; T, tenera; D, dura; CPO, crude palm oil; PKO, palm kernel oil; MY, Malaysia; PF, palm fruit; Msc, mesocarp; PKS, palm kernel shell; PK, palm kernel; PKC, palm kernel cake; RM, Malaysian Ringgit*.

## Discussion

Since the publication of the *E. guineensis* and *E. oleifera* reference genome sequences in Singh et al. (2013b), several critical milestones toward oil palm industry sustainability have been achieved. These advances include the identifications of the *SHELL* gene (Singh et al., 2013a), the *VIRESCENS* gene and mutations responsible for oil palm fruit color (Singh et al., 2014), as well as the *MANTLED* gene and the epigenetic abnormality responsible for the somaclonal mantling phenotype (Ong-Abdullah et al., 2015). These discoveries have introduced strategies for genetic or epigenetic testing impacting sustainability by ensuring that land devoted to oil palm production is optimally utilized. The present study will enhance the sensitivity of DNA-based screening for non-*tenera* contamination through the addition of three novel mutant alleles of *SHELL* to the two previously identified alleles (Singh et al., 2013a). These five mutations, clustering within a highly conserved 19 amino acid span of the MADS box domain, were sufficient to account for all *tenera* and *pisifera* phenotype palms within the diverse geography surveyed.

These findings, for the first time, also enabled a direct assessment of non-*tenera* contamination in independent planting sites and nurseries across Malaysia. Within these populations, contamination rates were substantially higher than the <1% contamination rate theoretically achievable through careful control of pollination (Corley, 2005). Although, contamination rates varied significantly both between surveyed regions (ranging from 2.5 to 30.0%) and between specific sites within those regions, no region was found to be free of contamination. Overall, the measured non-*tenera* contamination rate across all regions/sites on a weighted average basis was 10.9% (8.1% *dura* and 2.8% *pisifera*).

Given directly measured contamination rates among independent planting sites and nurseries, it is possible to model the real-world economic impact of the implementation of comprehensive *SHELL* genetic testing at the nursery stage in this sector, which would effectively eliminate the inadvertent planting of non-*tenera* materials. Economic modeling predicts that the comprehensive genetic testing to prevent cultivation of non-*tenera* palms in the independent sector alone would add ∼RM 1.05 BN ($300M USD) to Malaysian GNI annually, which represents RM 272 ($77.71 USD) in gains per screened palm. Screening would contribute RM 0.693 BN ($198M USD) to oil palm industry members annually representing RM 180 ($51.42 USD) per screened palm. Finally, *SHELL* gene testing would increase the Malaysian tax revenues annually by RM 0.26 BN (∼$75M USD) or RM 68 ($19.43 USD) for each palm screened. Therefore, *SHELL* genetic testing would have a major positive economic impact while improving oil palm sustainability by optimizing the utilization of existing planted area.

Although, over 10,000 palms and seedlings derived from multiple sites within each of 13 geographic regions were analyzed in the present study, it is noted that this survey of 36 independent planting sites and 21 nurseries represents only a small sampling of the over 200,000 independent planting sites throughout Malaysia. The findings reveal that non-*tenera* contamination is likely a concern throughout the entire palm industry, and they demonstrate that comprehensive genetic testing will have major positive environmental and economic impacts. However, substantially larger surveys of contamination, utilizing now available *SHELL* genetic testing, will be a critical area of future oil palm research.

## Author Contributions

E-TL, MA, RN, NL, JO, Ravigadevi Sambanthamurthi, and Rajinder Singh conceptualized the research program. LO, E-TL, MA, RN, MM, WO, AM, NL, JO, Y-MC, Ravigadevi Sambanthamurthi, and Rajinder Singh designed the experiments and coordinated the project. LO, NT, MAB, AVB, MB, ML, Y-MC, and Rajinder Singh conducted laboratory experiments. LO, E-TL, MA, NL, JO, MAB, AVB, NJ, MB, ML, SWS, Ravigadevi Sambanthamurthi, and Rajinder Singh assisted in data analysis. E-TL, JN, K-LC, MH, NA, and SWS performed bioinformatics analysis. LO, AF, AK, and SB coordinated collection of samples. RN, NL, AH-O, AN, HC, and SAS participated in the economic analysis. LO, E-TL, MA, RN, NL, JO, Ravigadevi Sambanthamurthi, and Rajinder Singh prepared and revised the manuscript. All authors approved the final manuscript.

## Conflict of Interest Statement

AK and SB are employees of Orion Biosains, a biotechnology company located in Puchong, Selangor, Malaysia that offers *SHELL* genetic testing to the oil palm industry under a royalty bearing license from the Malaysian Palm Oil Board. Orion Biosains is an affiliate of Orion Genomics, LLC based in St. Louis, MO. NL, JO, and AF are directors of Orion Biosains. NL, JO, AF, MAB, AVB, MB, ML, NJ, SWS, and CB are employees and stock holders of Orion Genomics, LLC. LO, E-TL, MA, RN, NT, JN, MM, K-LC, MH, NA, WO, AM, Y-MC, Ravigadevi Sambanthamurthi, and Rajinder Singh are employees of the Malaysian Palm Oil Board. LO, E-TL, MA, RN, SWS, NL, JO, AVB, MAB, Ravigadevi Sambanthamurthi, and Rajinder Singh are co-inventors on pending patents covering *SHELL* genetic testing.

## Footnotes

1 https://orionbiosains.com/products/2016-02-15-14-41-42/suresawit-shell/protocol/sybr-green-dye-detection

2 http://www.indexmundi.com/commodities/?commodity=palm-oil&months=360&currency=myr

3 http://www.indexmundi.com/commodities/?commodity=palm-kernel-oil&months=360&currency=myr
